# The dysregulation of leukemia inhibitory factor and its implications for endometriosis pathophysiology

**DOI:** 10.3389/fimmu.2023.1089098

**Published:** 2023-03-23

**Authors:** Katherine B. Zutautas, Danielle J. Sisnett, Jessica E. Miller, Harshavardhan Lingegowda, Timothy Childs, Olga Bougie, Bruce A. Lessey, Chandrakant Tayade

**Affiliations:** ^1^ Department of Biomedical and Molecular Sciences, Queen’s University, Kingston, ON, Canada; ^2^ Department of Pathology and Molecular Medicine, Kingston Health Sciences Centre, Kingston, ON, Canada; ^3^ Department of Obstetrics and Gynaecology, Kingston Health Sciences Centre, Kingston, ON, Canada; ^4^ School of Medicine, Wake Forest University, Winston-Salem, NC, United States

**Keywords:** leukemia inhibitory factor (LIF), endometriosis, vascularization, cytokine, immunomodulation, infertility

## Abstract

Endometriosis is an estrogen dominant, chronic inflammatory disease characterized by the growth of endometrial-like tissue outside of the uterus. The most common symptoms experienced by patients include manifestations of chronic pelvic pain- such as pain with urination, menstruation, or defecation, and infertility. Alterations to Leukemia Inhibitory Factor (LIF), a cytokine produced by the luminal and glandular epithelium of the endometrium that is imperative for successful pregnancy, have been postulated to contribute to infertility. Conditions such as recurrent implantation failure, unexplained infertility, and infertility associated diseases such as adenomyosis and endometriosis, have demonstrated reduced LIF production in the endometrium of infertile patients compared to fertile counterparts. While this highlights the potential involvement of LIF in infertility, LIF is a multifaceted cytokine which plays additional roles in the maintenance of cell stemness and immunomodulation. Thus, we sought to explore the implications of LIF production within ectopic lesions on endometriosis pathophysiology. Through immunohistochemistry of an endometrioma tissue microarray and ELISA of tissue protein extract and peritoneal fluid samples, we identify LIF protein expression in the ectopic lesion microenvironment. Targeted RT qPCR for LIF and associated signaling transcripts, identify *LIF* to be significantly downregulated in the ectopic tissue compared to eutopic and control while its receptor, *LIFR*, is upregulated, highlighting a discordance in ectopic protein and mRNA LIF expression. *In vitro* treatment of endometriosis representative cell lines (12Z and hESC) with LIF increased production of immune-recruiting cytokines (MCP-1, MCP-3) and the angiogenic factor, VEGF, as well as stimulated tube formation in human umbilical vein endothelial cells (HUVECs). Finally, LIF treatment in a syngeneic mouse model of endometriosis induced both local and peripheral alterations to immune cell phenotypes, ultimately reducing immunoregulatory CD206^+^ small peritoneal macrophages and T regulatory cells. These findings suggest that LIF is present in the ectopic lesions of endometriosis patients and could be contributing to lesion vascularization and immunomodulation.

## 1 Introduction

Endometriosis, a chronic inflammatory gynaecological disease, is defined by the growth of endometrial like tissue outside of the uterus (1). Lesions, referred to as ectopic tissue, can manifest throughout the abdominal cavity constituting the subtypes of endometriosis based on lesion placement and depth: superficial peritoneal, deep infiltrating, and ovarian (2). While lesion presentation provides a means for categorizing disease stage, there is currently no relationship between endometriosis subtype and patient symptomatology. Symptoms such as chronic pelvic pain, pain with urination, and infertility, vary across patients and disease stages (3, 4). Of note, infertility is experienced by approximately 30-50% of endometriosis patients (5). The cause and effect are still unclear surrounding the association between infertility and endometriosis, however there are numerous mechanisms that have been proposed. In this context, Leukemia Inhibitory Factor (LIF) has been implicated as a contributor to endometriosis associated infertility (6, 7).

LIF is a pleiotropic cytokine of the interleukin (IL)-6 family, with involvements in reproductive processes such as embryo implantation and decidualization, as well as regulation of the immune response. LIF is produced by the endometrial luminal and glandular epithelium during the mid to late secretory phase and is imperative for successful pregnancy through the orchestration of stromal cell decidualization (8, 9). Pivotal findings determined that LIF knock out mice were unable to support blastocyst implantation, implicating a critical role for LIF in fertility (10). As such, LIF mRNA and protein, both content and localization within the endometrium, have been studied in a variety of infertility cohorts including unexplained infertility (11, 12), recurrent implantation failure (13, 14), as well as in diseases associated with infertility such as adenomyosis (15, 16) and endometriosis (6, 7). Consistently it has been shown through immunohistochemistry (IHC) of the endometrium that the infertile cohorts- regardless of cause, have reduced endometrial LIF protein expression compared to fertile controls. This is in concert with cervical lavage samples from infertile adenomyosis patients obtained during the mid to late secretory phase, which had significantly lower LIF protein compared to fertile controls (15). In patients with mild to moderate endometriosis who are experiencing infertility, LIF expression was reduced in endometrial samples obtained during the mid-secretory phase, combined with elevated IL-6 and IL-1α in the peritoneal fluid (PF) (7). These inflammatory mediators were hypothesized to be a contributor to patient infertility yet the broader implications of LIF dysregulation within endometriosis pathophysiology have not been speculated upon and remain to be explored.

The role of LIF throughout the body is dynamic. In development, LIF is a regulator of embryonic stem cells, facilitating their pluripotency (17). In adults, LIF is produced in the uterus (18), lung (19), and central nervous system (20, 21), situated as a mediator between neuro-immune crosstalk and a regulator of anti-inflammatory pathways. The LIF receptor (LIFR) can be found on various cell types such as stromal, endothelial, epithelial, and immune cells- most notably T cells and macrophages (9, 22). However, the specific function of LIF is dependent on the local microenvironment, leading to both an inflammatory and anti-inflammatory response. LIF signaling activates three primary pathways known as- janus kinase (JAK)- signal transducer and activator of transcription (STAT3), mitogen activated protein kinase (MAPK), phosphatidylinositol-3 kinase (PI3k), which are associated with cellular proliferation and self-renewal (17). LIFs activation of these pathways has been demonstrated not only within reproductive processes and homeostatic immune regulation but additionally, within the pathological context of cancer. Specifically, breast (23–25), ovarian (26), pancreatic (27), and nasopharyngeal cancers (28), have demonstrated that LIF overexpression by tumours contributes to tumor growth and metastasis as mediated by the STAT3 pathway. Within these contexts, LIFs’ capacity to modulate immune phenotypes serves to promote immune evasion and treatment resistance. LIF has been shown to induce regulatory phenotypes in both myeloid and lymphoid cells, primarily working through alternatively activated macrophages (M2) to increase T regulatory (Treg) cell function (29–31). These mechanisms of tissue maintenance, growth, and immune evasion can be paralleled with endometriosis pathophysiology. Thus, LIFs production by ectopic tissue and potential contribution to lesion sustainment and immunomodulation warrants investigation.

To date, LIF has only been studied within the context of endometriosis-associated infertility however its potential contributions to disease pathophysiology remains to be understood. Through human patient data, endometriosis representative human cell lines, and a syngeneic mouse model of endometriosis, we demonstrate that LIF is present in the lesion microenvironment of endometriosis patients and could be contributing to endometriosis-associated lesion vascularization and immune dysregulation. These findings provide novel insight to the role of LIF within endometriosis.

## 2 Methods

### 2.1 Ethics statement

Ethics was approved for this study by the Health Sciences Research Ethics Board at Kingston Health Sciences Centre (KHSC), Queen’s University (Kingston, Ontario, Canada), Greenville Health System (Greenville, South Carolina, USA), the University of North Carolina at Chapel Hill (Chapel Hill, North Carolina, USA), and Wake Forest Baptist Health (Winston-Salem, NC, USA). Human ectopic and eutopic samples from endometriosis patients and control samples, from healthy fertile women, were collected as per institutional approved protocols and guidelines. Written, informed consent was acquired from all patients before acquisition and storage of samples.

### 2.2 Detection of LIF in endometriosis patient peritoneal fluid and tissue samples using ELISA

Endometriosis patient PF (n=5) and tissue protein extract (matched ectopic (n=13) and eutopic (n=12)) were analyzed to determine the concentration of LIF using an ELISA kit (BMS242, ThermoFisher). Protein extract and PF samples were obtained from separate endometriosis patient cohorts, resulting in n=17 samples. Briefly, 37mg of tissue was weighed and manually homogenized using ceramic power bead tubes (13113-50, QIAGEN) with the addition of tissue protein extraction reagent (78510, ThermoFisher) and protease inhibitor cocktail (535140, Sigma-Aldrich). Protein content was determined using a microplate bicinchoninic acid (BCA) protein assay kit (23532, ThermoFisher) and normalized to the lowest concentration (1202ug). For the ELISA, standards and samples were added to the plate and incubated at room temperature for 2hrs on a plate shaker. After incubation, the plate was washed with wash buffer before the addition of streptavidin and then incubated for 1hr in the same conditions. After washing, horseradish-peroxidase (HRP) was added, and the plate incubated for 30mins. Finally, a substrate solution of tetramethyl-benzidine was added and incubated for 10mins in the dark, after which stop solution was added. The plate was analyzed in a SpectraMax iD5 microplate reader (Molecular devices, California, USA) at an absorbance of 450nm with a reference wavelength of 620nm.

### 2.3 Immunohistochemistry for LIF on an endometrioma tissue microarray

A tissue microarray (TMA) was created with human patient samples from Kingston General Hospital as previously outlined (32). From a separate patient cohort, matched ectopic (endometrioma samples) and eutopic tissues collected from endometriosis patients (n=19) were compared to endometrium from healthy controls (n=22). Patients were identified as one of three menstrual states by pathologist review: proliferative, secretory, or inactive. The breakdown of patients by menstrual stage are as follows: Endometriosis (proliferative = 7, secretory = 8, inactive= 4), controls (proliferative= 18, secretory= 7, inactive= 1). For IHC, a 5µm section of the TMA was taken, subjected to xylene, and rehydrated with various concentrations of ethanol and citrisolv solutions. Antigen retrieval was performed with a citrate buffer for 20mins and subsequently stained with a polyclonal LIF antibody (26757-1-AP, ThermoFisher, 1:500) using a Leica Bond RX autostainer (Leica Biosystems- Microsystems Inc., IL, USA). The slide was scanned using an Olympus VS120 Virtual Slide Microscope (Olympus, USA) and analyzed using HALO image analysis software (Indica Labs, USA). Quantification of percent area positive for anti-LIF stain was performed on the total core area and the luminal and glandular epithelium respectively. As LIF is produced within the luminal and glandular epithelium, this delineation in area quantification provided a more accurate representation of LIF staining as cores differed in their stromal and epithelial composition.

### 2.4 Targeted RT qPCR array for LIF associated genes in endometriosis and control tissues

Total RNA was extracted from patient samples (n=8 matched endometriosis eutopic/ectopic tissues, n=9 healthy controls) using a total RNA isolation kit (17200, Norgen Biotek Corp., ON, Canada). Briefly, 20mg of tissue was added to a ceramic power bead tube (13113-50, QIAGEN, Hilden, Germany) with 600µL lysate buffer solution and digested using the Omni Bead Ruptor (PerkinElmer Comp., GA, USA). Samples were subsequently centrifuged at 10000g for 5min to pellet tissue debris. The resultant supernatant was aspirated and passed through a pre-assembled column to remove genomic DNA and collect total RNA. RNA was purified and reverse transcribed into complimentary DNA (cDNA) using a RT^2^ First Strand Kit (330401, QIAGEN). Quality of RNA and cDNA samples were tested using a Nanodrop 2000 Spectrophotometer (ThermoScientific, MA, USA). cDNA was used with targeted RT^2^ qPCR custom array plates (CLAH41769-(330171)), QIAGEN) to detect 19 key gene transcripts for downstream LIF family proteins and transcription factors, selected after extensive literature review. RT qPCR was conducted using the LightCycler 480 Real-Time PCR system (Roche Molecular Systems, Inc. Basel, Switzerland) with QuantiTect SYBR Green PCR mastermix (330501, QIAGEN). Relative gene expression values were calculated by delta delta CT method after normalization to housekeeping genes (*ACTB* and *GAPDH*). Primers for the following proteins and transcription factors were used: *LIF, LIFR, OSM, IL-6, IL6ST, CNTF, PRL, IGFBP-1, SOX2, SOCS3, JAK1, MAPK1, AKT, mTOR, POU5F1, PTPN11, PI3KR1, NANOG, STAT3.*

### 2.5 Human cell lines

Immortalized human endometriotic epithelial- 12Z cells (provided by Dr. Anna StarzinskiPowitz), human umbilical vein endothelial cells (HUVEC; CRL-1730, ATCC, VA, USA), and human endometrial stromal cells (hESCs; T0533 ABM, BC, Canada). Immortalized 12Z cells were maintained in DMEM/F-12 (11320033, ThermoFisher) supplemented with 10% fetal bovine serum (FBS; 97068-085, VWR), 1% penicillin/streptomycin (15140122, ThermoFisher) and 1% (100mM) sodium pyruvate (11360070, ThermoFisher). HUVEC cells were maintained with complete endothelial cell growth medium (211-500, Cell Application). hESCs were maintained in PriGrow (TM004, ABM) with 10% charcoal stripped FBS (12676029, ThermoFisher), 1% L-glutamine (A2916801, ThermoFisher) and 1% penicillin/streptomycin. All cell lines were cultured in T75 flasks and maintained until 70-80% confluence, with media changes every 2-3 days. Cells were kept in a humidified cell culture incubator at 37°C and with 5% CO_2_.

### 2.6 Multiplex cytokine analysis of endometriosis representative cell lines following rhLIF treatment

12Z, HUVEC, and hESCs were cultured in 24-well plates at 2.5x10^4^ cells/well. Cells were rested for 24hrs prior to treatment with PBS or rhLIF in the following concentrations: 1, 20, and 100ng/mL. Following a 24hr incubation period, cell supernatant was collected and stored at -80°C prior to multiplex cytokine analysis (HD-48 plex, EveTechnologies, AL, Canada). Exhaustive cytokine list as follows: sCD40L, EGF, Eotaxin, FGF-2, Flt-3 ligand, Fractalkine, G-CSF, GM-CSF, GROα, IFNα2, IFNγ, IL-1α, IL-1β, IL-1ra, IL-2, IL-3, IL-4, IL-5, IL-6, IL-7, IL-8, IL-9, IL-10, IL-12p40, IL-12p70, IL-13, IL-15, IL-17A, IL-17E/IL-25, IL-17F, IL-18, IL-22, IL-27, IP-10, MCP-1, MCP-3, M-CSF, MDC (CCL22), MIG, MIP-1α, MIP-1β, PDGF-AA, PDGF-AB/BB, RANTES, TGFα, TNFα, TNFβ, VEGF-A.

### 2.7 Proliferation and apoptosis assays in endometriosis representative cell lines treated with rhLIF

Cell proliferation and apoptosis were measured in 12Zs, HUVECs, and hESCs following treatment with PBS or varying concentrations of recombinant human LIF (rhLIF; 7734-LF, R&D Systems, MN, USA). Briefly, cells were seeded at 5x10^3^ cells/well in 96 well plates (hESCs and 12Zs used phenol red free DMEM F-12 (21041025, ThermoFisher) and rested for 24hrs. Media was then changed with media containing either PBS or rhLIF at 1,20, or 100ng/mL, and incubated for an additional 24hrs. Proliferation was determined using a WST-1 assay (0501594400, Sigma-Aldrich, MO, USA) and apoptosis determined using Caspase Glo 3/7 reagent (G8091, Promega, WI, USA). Briefly, 10µL of WST-1 reagent was added per well to achieve a 1:10 dilution before a 2hr incubation at 37°C. To determine apoptosis, 100µL of Caspase Glo 3/7 reagent was added per well to achieve a 1:1 dilution before incubation at room temperature for 2hrs. A SpectraMax iD5 microplate reader (Molecular Devices) was used to obtain the absorbance and luminescence for the proliferation and apoptosis assays respectively. The absorbance of formazan dye produced during the WST-1 reaction was recorded at 450nm with a reference wavelength of 650nm. Each proliferation and apoptosis experiment was repeated at least 3 times per individual cell line, thus data shown is representative.

### 2.8 Endothelial tube formation assay

HUVEC cells were utilized for a tube formation assay as per the protocol of the manufacturer in a µ-slide assay format. Briefly, IBIDI microplates (81506 Ibidi, Germany) were loaded with 10µL of Matrigel™ (354230, Corning, USA) and incubated for 1hr to allow for Matrigel™ polymerization. HUVEC cells were plated in triplicates at 1x10^4^ cells/well above the polymerized Matrigel™ in 50µL of media with one of the following treatments: VEGF, PBS, LIF (1, 20, 100ng/mL). Cells were incubated at 37°C and with 5% CO_2_ for 4hrs. Images were taken on a Nikon TE200 inverted epifluorescence microscope using a 10× objective and a cooled CCD camera and analyzed by WimTube: Tube Formation Assay Image Analysis Solution (33).

### 2.9 Murine model of endometriosis

Seven-to-eight-week-old female C57BL/6 mice (n=17; Charles River Laboraties, MA, USA) were housed in conventional housing with an automated watering system and 12-hr light-dark cycle at 3-4 animals per cage. To induce endometriosis, uterine horns were harvested from donor mice (n=3) and dermal biopsy punches (3mm^3^) were used to obtain uterine fragments to be explanted into recipient mice. For surgery, mice were anesthetized with 2.5% isofluorane. Briefly, an incision was made into the abdomen to allow access to the peritoneum, upon which two 3mm^3^ uterine fragments were attached with Vetbond adhesive (1469SB, 3M, MN, USA) to the peritoneal wall. A suture and two staples were used to close the peritoneum and skin respectively. To understand the influence of LIF on endometrial lesion growth and immune cell populations, mice received daily intraperitoneal (i.p) injections of either PBS (control; n=6) or recombinant mouse LIF (rmLIF; n=6; 8878-LF-100/CF, R&D Systems) for 14 days. This experiment was duplicated with varying rmLIF doses (300ng and 1µg). On day 14, animals were sacrificed and peritoneal lavage was performed with ice-cold PBS before the spleen, uterine horns, and endometriosis-like lesions were excised. Lesions were placed in 4% paraformaldehyde and kept in 4°C for 24hrs. Fixed lesions were then transferred to 70% ethanol before processing for paraffin embedding. PF and splenocytes were used for flow cytometry. Spleens were excised and immediately placed in RPMI 1640 supplemented with 5% FBS. To isolate splenocytes, spleens were mechanically digested through a 70µm strainer and centrifuged at 300g for 5min at 4°C. Both PF and splenocytes were pelleted and resuspended in FBS with 10% dimethyl sulfoxide (DMSO; Sigma-Aldrich) for cryopreservation.

### 2.10 Flow cytometry

Mouse PF and splenocytes were thawed in a water bath at 37°C and reconstituted in 15mL of FACs buffer (PBS with 10% FBS). Cells were centrifuged at 300g for 5min 4°C and supernatant decanted. Following the addition of DNAse 1 (10104159001, Millipore Sigma; 100µg/µL) samples were incubated for 10min at 4°C as per manufacturers guidelines. Cells were neutralized with 10mL of FACS, centrifuged at 300g for 5min 4°C, then resuspended for cell counting by an automated cell counter (Countess 3, ThermoFisher). Samples of 5x10^5^ cells were used for staining for flow cytometry. To limit non-specific antigen binding, samples were stained with anti-mouse TruStainFcX (101320, BioLegend; 1:50) and incubated for 10min at 4°C. Subsequently, extracellular staining for myeloid and lymphoid markers was performed with a 30min incubation period. All products were obtained from BioLegend unless otherwise stated: fixable viability dye, eFluor780 (65-0865-14, ThermoFisher; 1:500), Brilliant Violet (BV)510-anti-CD3 (100234; 1:40), BV785-anti-CD4 (100552; 1:80), BV605-anti-CD8 (100744; 1:40), FITC-anti-CD25 (102005; 1:50), PB- anti-CD11b (1012224; 1:50), PE-Cy7-anti-F4/80 (123114; 1:80), AF700-anti-MHCII (107621; 1:200). Cells were fixed and permeabilized using a FOXP3 Fixation and Permeabilization Kit (00-5523-00, eBioscience) following the manufacturers recommendation. After permeabilization, cells were stained intracellularly with PE-anti-FOXP3 (126404; 1:20) and APC-anti-CD206 (141708; 1:40). Following a 30min staining incubation, cells were washed with FACS.

All data was acquired on the CytoFLEX S flow cytometer (Beckman Coulter, CA, USA) and analyzed using FlowJo software (version 10). Half-heat killed cells were used to detect viability and fluorescence minus one (FMO) controls used to set positive population gates.

### 2.11 Immunohistochemistry of mouse lesions

Paraffin embedded blocks were sectioned to 5µm thickness and subjected to deparaffinization with xylene before rehydration with various concentrations of ethanol and citrisolv solutions. Antigen retrieval was performed with citrate buffer for 20mins and subsequent staining with polyclonal antibodies for mouse Ki67 (ab15580, Abcam, 1:1000), CD31 (77699S, New England Biolab, 1:100), LIF (PA5-115510, Invitrogen, 1:50), and LIFR (101228, Abcam ab, 1:2000) were completed using a Leica Bond RX autostainer. Lesions were analyzed using a singular computer-generated algorithm created for each stain (Ki67, CD31, LIF, LIFR). Percent area quantification for positive stain was used for CD31, LIF, and LIFR. A cytonuclear algorithm was used to detect individual cell expression of Ki67 after which the percent of proliferating cells (Ki67^+^) could be expressed as a percent of the total cells. Slides were scanned using an Olympus VS120 Virtual Slide Microscope (Olympus) and image analysis performed using HALO image analysis software (Indica Labs).

### 2.12 Statistics

All statistical analyses were performed on GraphPad Prism9 (CA, USA). Unpaired students T-test used for analysis between two groups, and one way-ANOVA with Tukey *post-hoc* used for multiple group comparisons. A p value equal or less than 0.05 was considered statistically significant.

## 3 Results

### 3.1 LIF is present in the ectopic lesions of endometriosis patients and is dysregulated across the ectopic and eutopic tissues

**Figure 1:**
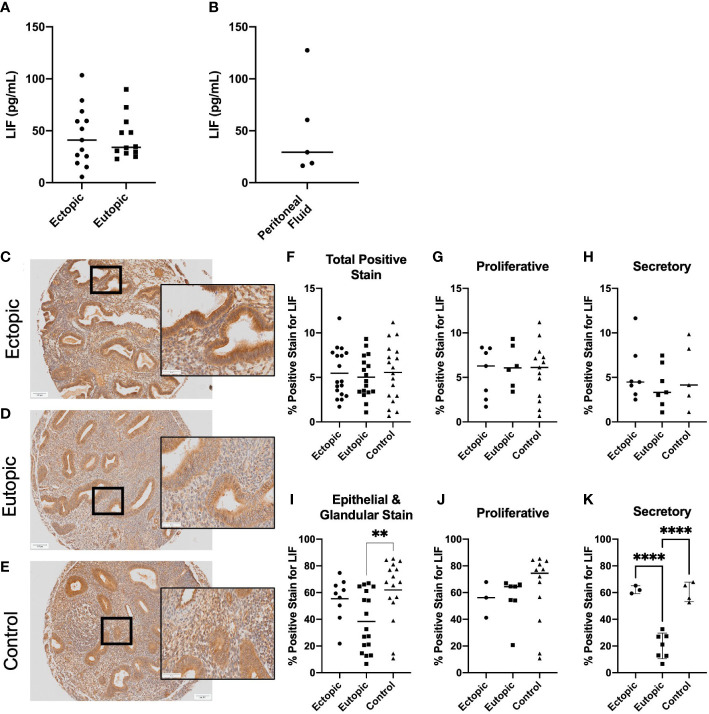
LIF is present in the ectopic lesion microenvironment of endometriosis patients. Ectopic and eutopic tissues **(A)** and PF **(B)** from endometriosis patients contain LIF as detected through ELISA. No significant differences in LIF values were seen across tissue type. Analysis performed as unpaired Student’s T-test. **(C–E)**, Endometrioma TMA of matched endometriosis (eutopic and ectopic; n=19) and control endometrium (n=22) was stained with an anti-LIF antibody. Area quantification of percent positive stain was calculated for the total core area **(F–H)** and luminal and glandular epithelium **(I–K)** respectively. Patients were stratified by menstrual phase- proliferative **(G, J)** and secretory **(H, K)**, for both area quantifications. Patient samples used in **(A–C, E)** reflect 3 separate patient cohorts. Magnification provided at 4x and 20x; scale bar 100µm. Analysis performed as one-way ANOVA with Tukey post-hoc, **P<0.01, ****P<0.0001.

Previous reports have analyzed LIF within the endometrial tissue of endometriosis patients as it pertains to fertility status, however it has yet to be identified within the ectopic lesion. To gain insight into the presence of LIF within the lesion microenvironment, endometriosis patient tissues and PF samples were analyzed for LIF by ELISA. LIF was detected in all tissue extracts (**Figure 1A** **)** and PF samples (**Figure 1B** **)** at varying levels; ectopic= 45.08 ± 28.34, eutopic=43.52 ± 20.87, PF=50.49 ± 46.48. No significant differences in protein expression were found between the eutopic and ectopic tissues of endometriosis patients. PF samples from healthy, fertile controls could not be obtained due to logistical difficulties; however, demonstrating the presence of LIF in the PF of women with endometriosis represents a novel finding in and of itself as it demonstrates that LIF is present in the endometriotic microenvironment.

Our next step was to gain spatial understanding of LIF within the lesion microenvironment, thus, IHC was performed on a TMA of endometrioma and control endometrium samples (endometriosis matched; n=19, controls; n=21) (**Figures 1C–K**). When specified to the epithelium, eutopic tissues demonstrated significantly less percent area positive for LIF stain than the control (p=0.0096), as previously noted in literature (**Figure 1I**). Percentage positive for LIF stain was elevated in ectopic lesions compared to eutopic but was not statistically significant. However, when patients were stratified by menstrual phase, there was a significant difference between ectopic and eutopic LIF staining (p<0.001) (**Figure 1K** **)** illustrating a dysregulation in the production of LIF within endometriosis patients during the secretory phase.

**Figure 2:**
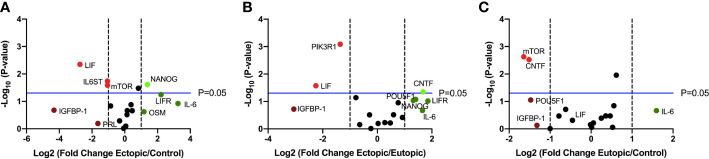
LIF gene expression is significantly downregulated in ectopic tissue compared to eutopic and control. Volcano plots showing differentially expressed genes in the LIF signaling pathway between **(A)** ectopic (n=9) and control (n=10), **(B)** ectopic and eutopic (n=9), **(C)** eutopic and control tissue samples. Vertical dashed lines indicate a fold change of +/-1 and horizontal blue line denotes a significance value of P=0.05.

Finally, we performed targeted RT qPCR using a custom array with select genes involved in the LIF signaling pathway and including members of the IL-6 family of cytokines, to identify differentially expressed transcripts (**Figure 2**). Of the 19 genes studied, *LIFR, IL-6, NANOG* were upregulated and *LIF, IGFBP-1* were downregulated in the ectopic tissue, compared to the eutopic endometrium from endometriosis patients and healthy controls (**Figures 2A, B**).

### 3.2 LIF treatment promotes the production of immune recruiting cytokines and induces tube formation in human umbilical vein endothelial cells

**Figure 3:**
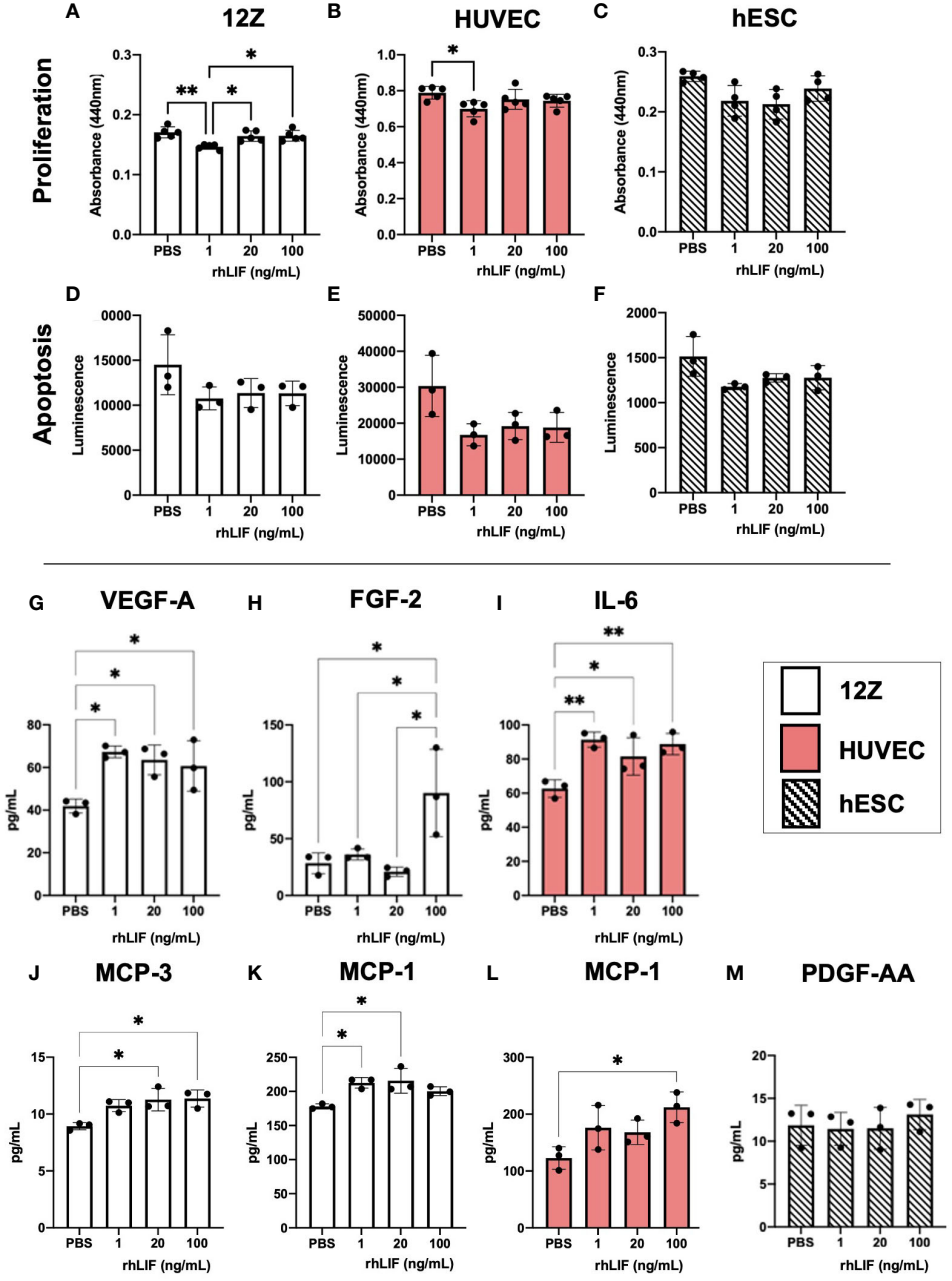
LIF treatment *in vitro* did not alter proliferation or apoptosis in endometriosis representative cell lines but stimulated the release of growth factors and immune recruiting cytokines. WST-1 **(A–C)** and Caspase **(D–F)** assays were conducted in endometriosis representative cell lines- 12Zs (white bars), HUVECs (red bars), and hESCs (dashed bars), to detect LIF influence on proliferation and apoptosis respectively. **(G–M)**, Cell lines were treated for 24hrs with PBS or varying rhLIF concentrations (1, 20, 100ng/mL) and supernatant analyzed for 48 cytokines pertaining to angiogenesis, inflammation, and cell growth (HD48-Multi-plex Analysis, EveTech). Analysis performed as one-way ANOVA with Tukey *post-hoc*, *P<0.05, **P<0.01.

LIF is a known immunomodulator, working as both an inflammatory and anti-inflammatory cytokine depending on the microenvironment. Additionally, as LIF can promote vascularization and proliferation, we sought to determine the effects of LIF on endometriosis lesion representative cell lines. We used 12Zs- an endometriotic epithelial cell line, and hESCs- a human endometrial stromal cell line, to represent the two primary cellular components of the endometrium and ectopic tissue being epithelial and stromal cells. Additionally, we utilized HUVECs- human umbilical vein endothelial cells, as they are a well-established model for angiogenesis. All cell lines were treated with varying rhLIF concentrations (1, 20, 100ng). Proliferation and apoptosis were measured using a WST-1 and caspase 3/7 glo assay respectively. LIF treatment did not result in detectable proliferation in any of the cell lines, but rather at the lowest dose decreased proliferation compared to the PBS control (**Figures 3A–C**). This reduced proliferation was not attributed to apoptosis, however, as the caspase assay showed no alterations to apoptosis regardless of the dose of LIF treatment (**Figures 3D–F**).

Cell supernatant, collected in response to rhLIF treatment (1, 20, 100ng/mL), was analyzed using a multiplex cytokine array for predominant pro-inflammatory/immunoregulatory cytokines, chemokines, and growth factors (**Figures 3G–M**). Significant production of immune recruiting cytokines such as monocyte chemoattractant protein (MCP)-1 and MCP-3 were produced in 12Zs and HUVECs upon LIF treatment (**Figures 3J–L**), but not in hESCs. Further, 12Zs secreted significantly higher concentrations of vascular endothelial growth factor (VEGF) with all treatment doses compared to PBS, indicating LIF as a potential promoter of angiogenesis *in vitro* (**Figure 3G**). Additional cytokines detected in the cell supernatants can be found in **Supplemental Figure 1**.

**Figure 4:**
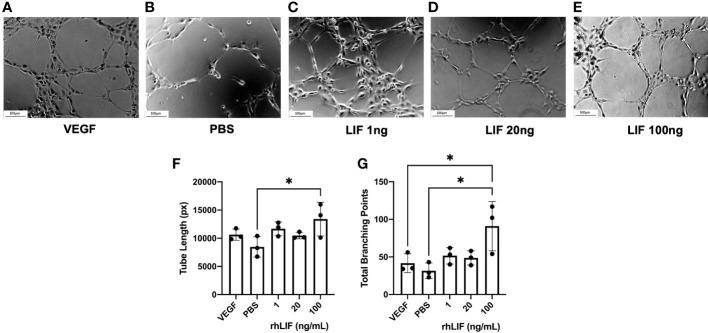
LIF treatment promotes tubulogenesis in HUVEC cell line. HUVEC were treated with VEGF, PBS, or rhLIF (1, 20, 100ng/mL) and incubated for 4hrs before image acquisition- representative images provided for each treatment condition **(A–E)**. Images were analyzed by WIMASIS Software to determine metrics of tube formation including **(F)** tube length and **(G)** total branching points. Analysis performed as one-way ANOVA with Tukey post-hoc, *P<0.05. Scale bar 100µm.

To determine LIFs influence on angiogenesis, a tubulogenesis assay was performed with HUVECs (**Figure 4**). Significantly elevated tube length (p<0.05) and number of total branching points (p<0.05) were seen with the 100ng rhLIF treatment compared to the PBS control (**Figures 4F, G** **)**.

### 3.3 LIF treatment in a syngeneic mouse model of endometriosis alters the local and peripheral immune response

**Figure 5:**
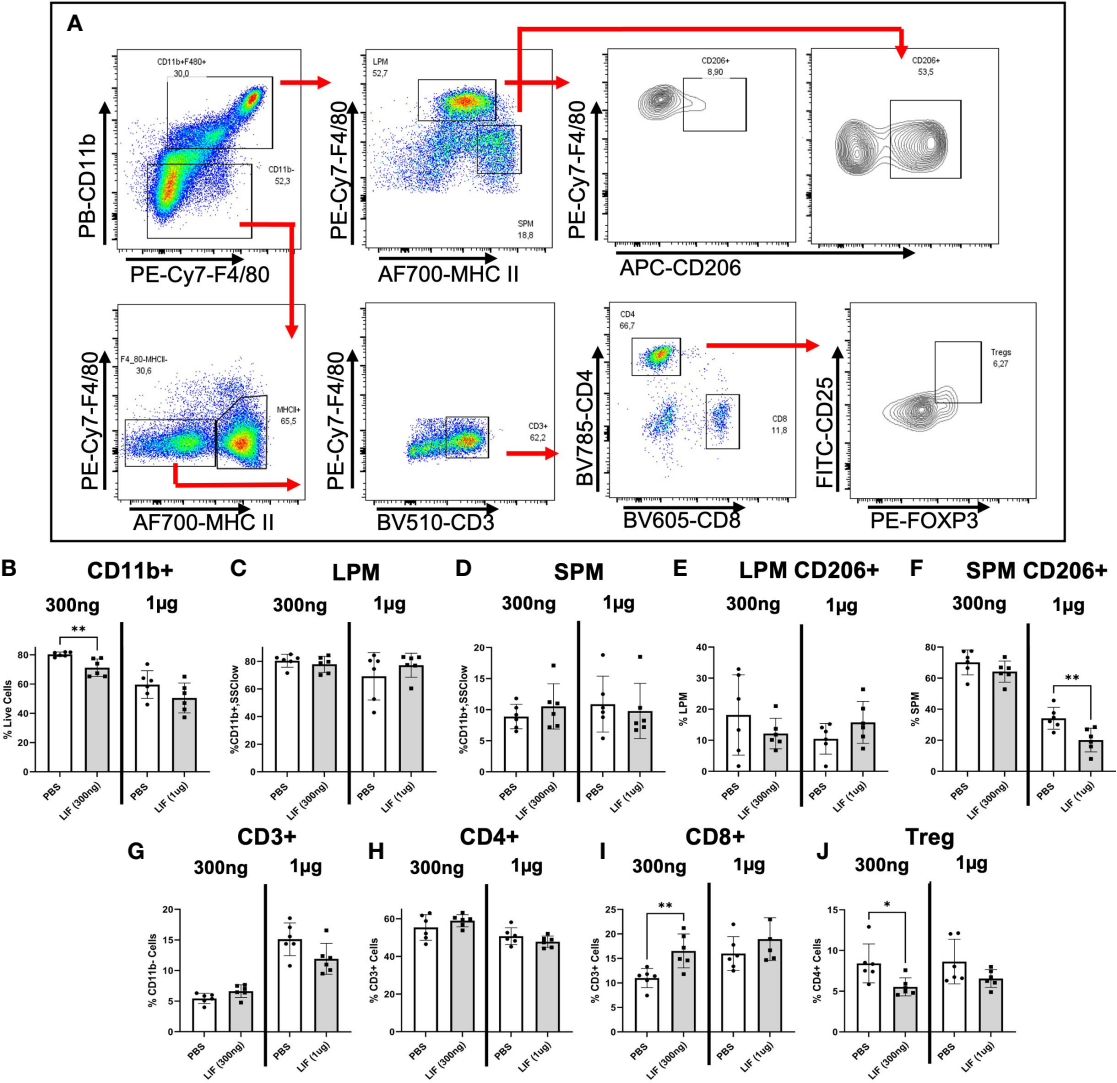
LIF treatment in a mouse model of endometriosis alters the local peritoneal immune response. **(A)** Gating strategy for flow cytometric analysis of myeloid **(B–F)** and lymphoid **(G–J)** markers on immune cells from the PF of mice injected i.p with PBS (white bars) or rmLIF (grey bars; 300ng, 1*µ*g) for 14 days. LPM gated as: single cells, live, SSC^low^, CD11b^+^, F4/80^hi^, MHCII^low^. SPM gated as: single cells, live, SSC^low^, CD11b^+^, F4/80^mid^, MHCII^hi^. Tregs gated as: single cells, live, SSC^low^, CD11b^-^, F4/80^-^, MHCII^-^, CD3^+^, CD4^+^, CD25^+^, FOXP3^+^. Results reflect duplicate experiments- one per rmLIF dosage. Analysis performed as unpaired Student’s T-test, *P<0.05, **P<0.01. LPM, large peritoneal macrophages, SPM, small peritoneal macrophages.

**Figure 6:**
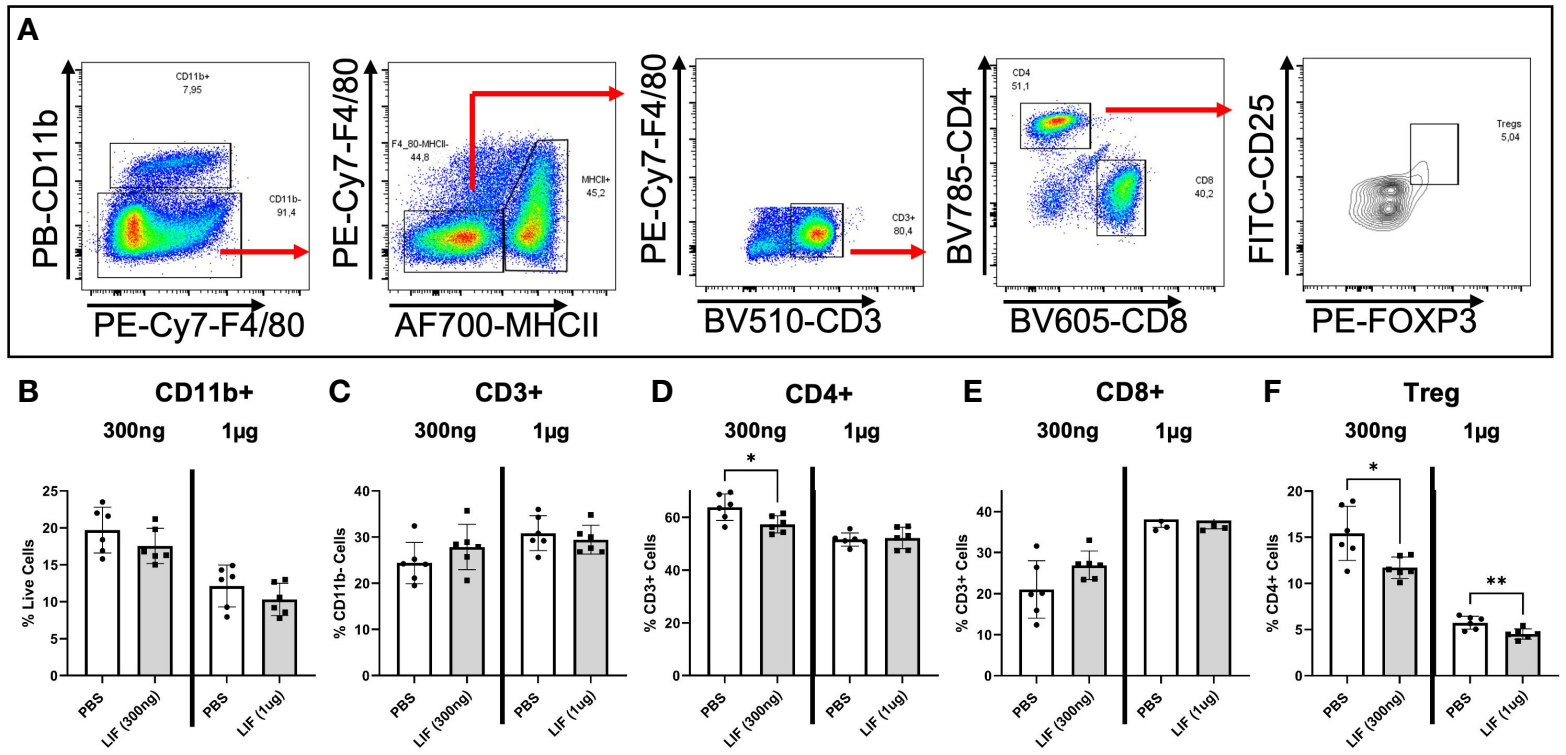
LIF treatment in a mouse model of endometriosis alters the peripheral immune response. **(A)** Gating strategy for flow cytometric analysis of myeloid **(B)** and lymphoid markers **(C–F)** on immune cells from the spleen of mice injected i.p with PBS (white bars) or rmLIF (grey bars; 300ng, 1µg) for 14 days. Tregs gated as: single cells, live, SSC^low^, CD11b^-^, F4/80^-^, MHCII^-^, CD3^+^, CD4^+^, CD25^+^, FOXP3^+^. Results reflect duplicate experiments- one per rmLIF dosage. Analysis performed as unpaired Student’s T-test, *P<0.05, **P<0.01.

To understand the influence of LIF on immune cell recruitment and polarization, we surgically induced endometriosis in C57BL/6 mice and performed daily i.p. injections of recombinant mouse LIF [rmLIF; 300ng and 1µg, based on available literature (28, 30)] or PBS for 14 days. To capture alterations to the local and peripheral immune response, PF (**Figure 5**) and splenocytes (**Figure 6**) were harvested for flow cytometric analysis of myeloid and lymphoid immune cell subsets. With LIF treatment, regardless of dose, there was a reduction in immunoregulatory phenotypes in the PF and spleen. In the PF, CD206^+^ small peritoneal macrophages (SPMs; gated as: singlet, live, side scatter (SSC)^low^, CD11b^+^, F4/80^mid^, MHCII^hi^, CD206^+^) and Treg (gated as: singlet, live, SSC^low^, CD11b^-^, F4/80^-^, MHCII^-^, CD3^+^, CD4^+^, CD25^+^, FOXP3^+^) cells were significantly decreased compared to PBS control (**Figures 5E,J**). Similarly in the spleen, Treg cells were significantly reduced with LIF treatment, as were CD4^+^ cells (**Figures 6D, F**). Of note, the PF of mice receiving the low LIF dose had significantly more CD8^+^ T cells compared to PBS (p<0.01) (**Figure 5I**).

### 3.4 LIF treatment did not alter lesion associated proliferation or angiogenesis in a syngeneic mouse model of endometriosis

**Figure 7:**
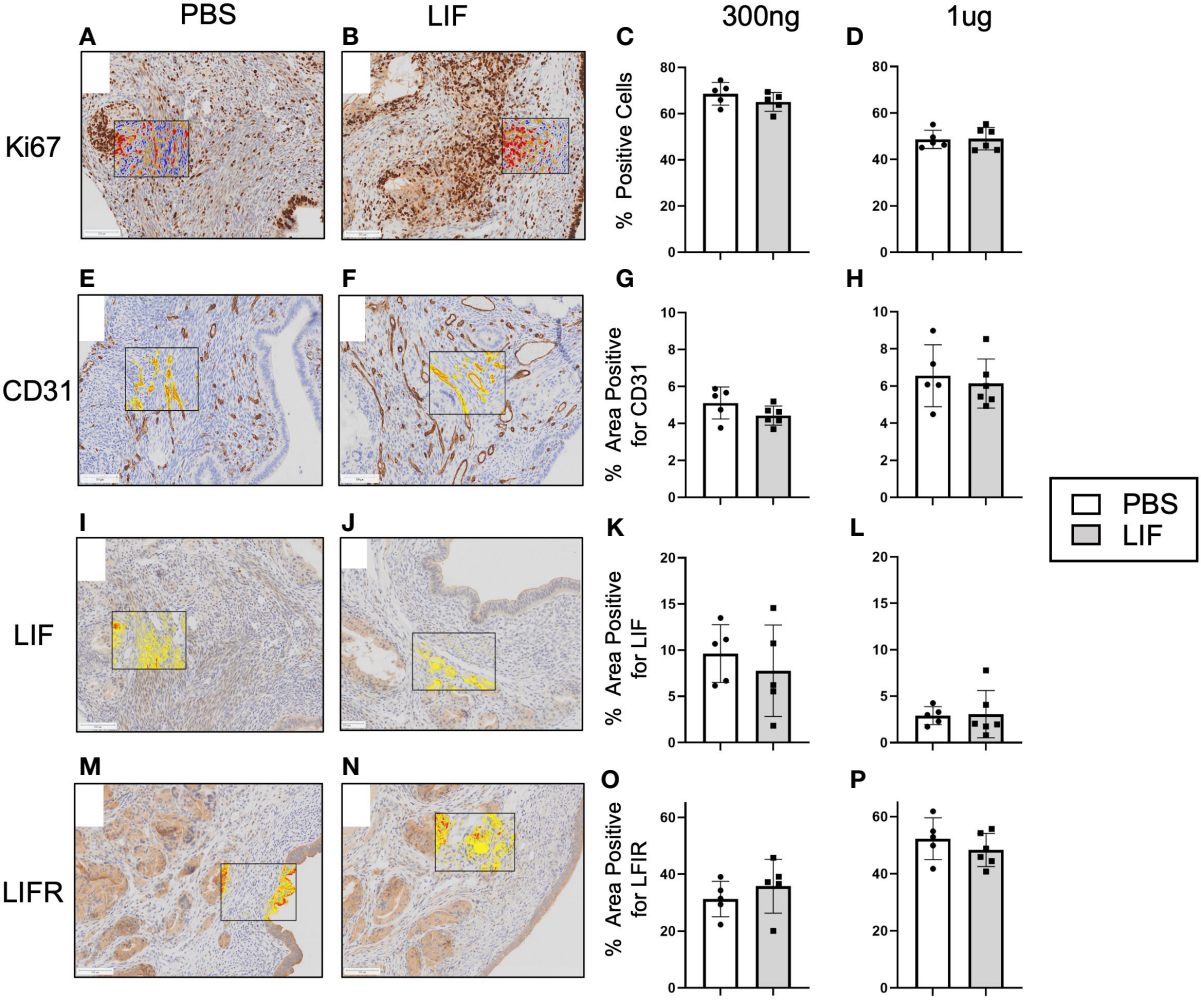
LIF treatment did not alter lesion growth or proliferation in a mouse model of endometriosis. I.p injections of PBS (white bars) or rmLIF (grey bars; 300ng, 1µg) were administered to C57BL/6 mice (n=6 for all groups) for 14 days, one week after endometriosis inducing surgery. Endometriosis-like lesions were collected upon sacrifice and subjected to IHC for markers of proliferation-Ki67 **(A, B)** and angiogenesis-CD31 **(E, F)**, as well as LIF **(I, J)** and LIFR **(M, N)**. Representative stain analysis provided for each marker from both the PBS and LIF treatment groups. Ki67 was analyzed as percent of cells expressing Ki67 over the total cell number as detected by a cytonuclear algorithm **(C, D)**, while all other stains (CD31, LIF, LIFR) were analyzed by percent area quantification of stain **(G, H, K, L, O, P)**. No statistical differences were detected across the four stains. Analysis performed as unpaired Student’s T-test. Scale bar 100µm.

LIF has been shown to promote tumor growth and vascularization in cancer, thus we sought to determine if those effects were withstanding in our syngeneic mouse model of endometriosis. Lesions harvested from LIF (300ng and 1µg) and PBS treated mice were fixed in paraformaldehyde and embedded in paraffin for IHC. Lesions were stained for markers of angiogenesis- CD31 and proliferation- Ki67, as well as LIF and LIFR (**Figure 7**). No differences were detected in any of the markers regardless of LIF dosage.

## 4 Discussion

Foundational knowledge of LIF is centered on its role in maintaining embryonic stem cell pluripotency (17), facilitating successful pregnancy through stromal cell decidualization and trophoblast implantation (8, 34), and participation in neuroimmune modulation (21). These situate LIF as a primary regulator of various homeostatic and pathologic signaling pathways within the body. Recent cancer literature has revealed that LIF is a contributor to immune evasion and facilitation of tumor growth and metastasis (28, 35). In endometriosis, LIF has only been investigated for its contributions to infertility (6, 7) however its presence within the lesion microenvironment and impact on lesion maintenance and the immune contexture remained unexplored.

Our findings identify LIF in the lesion microenvironment, with detection of LIF in the PF and protein extracts of both ectopic and eutopic endometriosis tissues. Spatial localization of LIF in endometriosis patients and control tissues, as provided by our IHC data, further corroborates that LIF is present in ectopic lesions with LIF staining localized to the luminal and glandular epithelium. Across both the proliferative and secretory phases, LIF expression by the ectopic luminal and glandular epithelium is similar to that of the control endometrium. Interestingly, within the secretory phase, ectopic LIF expression is significantly greater than eutopic. This reduced eutopic LIF expression is in accordance with infertility research (7), demonstrating that the endometrium of endometriosis patients has aberrant LIF expression during the secretory phase. Together, these results indicate that ectopic LIF production is rescued in endometriosis patients, highlighting a dysregulation of LIF between endometriosis tissues. The mechanism behind this recued phenotype requires further investigation as does the source of LIF production within the lesion microenvironment. LIF is produced by a variety of cell types including but not limited to endometrial luminal and glandular epithelium, endothelial cells (36), macrophages, and T cells (21). As patient PF and tissue protein extract consist of contributions from heterogenous cell types, continued investigation into the primary contributors of LIF to the lesion microenvironment is needed.

Targeted RT qPCR data provides further insight into LIF production within ectopic lesions. Our findings revealed that *LIF* gene expression is significantly downregulated while its primary receptor *LIFR* is upregulated in the ectopic tissue compared to eutopic and healthy control samples. This suggests that ectopic tissue is likely receptive to LIF but not producing it, potentially due to the high levels of LIF detected within the lesion microenvironment. In support of LIF signaling, *NANOG*, a downstream transcription factor of LIF associated with the maintenance of cell stemness, was significantly upregulated in the ectopic tissues compared to control and upregulated, though not significantly, compared to eutopic tissue. NANOG production is present in embryonic stem cells to maintain cell pluripotency and if present in adult tissues, serves as an oncogene, contributing to tumorigenesis (37). Our findings are in accordance with other endometriosis literature identifying downstream LIF targets NANOG, OCT-3/4, and SOX2 to be elevated in ectopic lesions compared to control endometrium (38, 39). These studies however do not mention LIF as a mediator of these pathways, thus our findings offer a novel perspective to view the activation of these stemness transcription factors within endometriosis. It is notable however, that NANOG can be regulated in the absence of LIF. E-cadherin can signal through STAT3 to increase NANOG transcription, while p53 can inhibit NANOG transcription through binding of its promoter region (37, 40). Thus, further investigation is required to confirm whether the upregulation of *NANOG* is due specifically to LIF signaling or other mediators in endometriosis.

Additionally, it is worth noting that other IL-6 family proteins (such as ciliary neurotrophic factor (CNTF) and oncostatin-M (OSM)) use the LIF receptor for signaling and have been implicated in various aspects of endometriosis. CNTF has been investigated for its potential association with sensitization and pain (41, 42), while OSM has been shown to inhibit endometrial stromal cell growth, with endometriotic stromal cells being resistant to this inhibitory effect (43). Our customized RT qPCR array included both CNTF and OSM to determine whether IL-6 family proteins or LIF specifically were dysregulated within endometriosis. Our findings demonstrate a significant upregulation of CNTF within the ectopic tissue compared to eutopic and significant downregulation in the eutopic tissue compared to the control. While OSM is upregulated, though not significantly, within the ectopic tissue compared to control. These findings suggest that the IL-6 family proteins are dysregulated within the ectopic tissue, with further research needed to determine the impact on lesion sensitization and growth.

LIF has been shown to promote tumor growth and metastasis through carcinoma cell proliferation, however in our *in vitro* models there were limited alterations to proliferation across various cell types- 12Z, hESC, and HUVEC. Yet treatment of these endometriosis representative cell lines with rhLIF yielded the production of immune recruiting and inflammatory cytokines MCP-1, MCP-3, and IL-6, as well as the angiogenic factor VEGF. The role of LIF as an angiogenic factor is contentious throughout the literature, reflecting the nuances through which microenvironments modulate LIF signaling. LIF has been demonstrated to regulate vascularization in concert with oxygen availability, meaning that it can prevent or promote the formation of blood vessels as seen in mouse models of ocular vascularization (44). Our findings, suggest that LIF is a promoter of angiogenesis both indirectly through the promotion of the angiogenic factor VEGF from 12Zs and directly by increasing endothelial tube formation in HUVECs. We sought to visualize these effects of LIF treatment within our mouse model through examination of indirect alterations to lesion architecture including proliferation and angiogenesis, however no significant changes were found. This discordance in angiogenesis can in part be attributed to temporal variations in experimental end points, where the tubulogenesis assay demonstrated short term response and our mouse model captures a more chronic response. Additionally, these are varying endothelial cell types, thus further investigation of LIF specifically on endometriotic endothelial cells is needed to elucidate its role within endometriosis.

LIFs role as an immunomodulator is co-opted in pathologies like cancer, as the immunoregulatory environment perpetuates tumor immune evasion and promotes resistance to treatment (23, 28). As our human data highlights the presence of LIF in the lesion microenvironment and our *in vitro* evidence suggests a role in angiogenesis and immune recruitment, we wanted to understand the impact of elevated LIF on lesion development and the immune contexture in our syngeneic immunocompetent mouse model of endometriosis. LIF treated mice did not demonstrate an increased number of infiltrating immune cells, but the composition of immune cell phenotypes was altered both locally and systemically. At both a low (300ng) and high (1µg) dose of rmLIF, there were significant reductions in immunoregulatory myeloid and lymphoid phenotypes, mainly CD206^+^ SPMs and Tregs. Further, CD8^+^ T cells were upregulated in the PF of the low dose rmLIF treated group. LIF has been demonstrated to assist in polarizing macrophages to an M2 phenotype and works through these cells to increase Treg functioning (29–31). Further, LIF provides barriers to CD8^+^ T cell infiltration due to its influence on M2 macrophages. In a mouse model of breast cancer, LIF was shown to operate through M2 like- tumor associated macrophages to silence CD8^+^ T cells *via* epigenetic modification (30). Our results appose these findings, potentially providing insight into a novel endometriosis associated LIF pathway whereby LIF intervention is reducing immunoregulatory phenotypes and promoting a potential cytotoxic response. Further phenotypic characterizations are needed to clarify the activation status of the CD8^+^ T cells present. Notably IL-6 has been identified as a key factor in assisting LIF polarization of macrophages to an M2 phenotype (26). Multiplex cytokine analysis of the PF demonstrated undetectable or negligible levels (<4pg/mL) of IL-6 (data not shown) suggesting that IL-6 was not produced in sufficient quantities in our mouse model with this treatment and time course for macrophage polarization to occur. Finally, direct comparison between rmLIF doses was not possible due to batch effect variations. Despite this limitation, the trend of reduced immunoregulatory phenotypes was consistent between LIF treatments supporting its role as an immunomodulator within endometriosis.

While we provide previously unexplored dimensions of LIF in endometriosis pathophysiology beyond infertility, we acknowledge some of the limitations of the work that are inherent to endometriosis research. Access to representative patient samples from each disease subtype and severity is limited. Additionally, as most patients have irregular menstrual cycles, there are limitations to identifying specific occurrences within defined menstrual phases. Due to limited access to PF samples from healthy controls only endometriosis LIF expression in the PF was shown. While we present this data to document LIF presence in endometriosis PF samples, future studies are needed to provide a comparison between endometriosis and control samples. Further, infertility is not a symptom that all patients experience yet it is one that is common, being present in around 30-50% of cases (5). Within our patient samples fertility status was not known preventing us from including this as a factor within our analysis. Thus, continued investigation is needed to determine whether LIF dysregulation is specific to endometriosis patients with infertility or whether it can be found in fertile patients as well. Finally, as the endometrioma TMA data was more conclusive for LIF presence within the ectopic tissue than the ELISA data (which contained mixed endometriosis subtypes), perhaps the type of endometriosis is a factor in the degree of LIF dysregulation, thus further investigation within endometriosis subtypes is needed.

In conclusion, this study demonstrates that LIF is present in ectopic endometriosis lesions and provides insight to the potential contributions it has to endometriosis pathophysiology. While it is known that some endometriosis patients experience alterations to eutopic LIF production, it is still not known whether this is a consequence of endometriosis associated infertility or whether this contributes to endometriosis pathophysiology. Ultimately, further investigation into the role of LIF across endometriosis subtypes and stages is required to better address its role within both endometriosis-associated infertility and pathophysiology.

## Data availability statement

The original contributions presented in the study are included in the article/**Supplementary Material**. Further inquiries can be directed to the corresponding author.

## Ethics statement

The studies involving human participants were reviewed and approved by Health Sciences Research Ethics Board at Kingston Health Sciences Centre, Queen’s University Health Sciences Research Ethics Board, Greenville Health System, and Wake Forest Baptist Health. The patients/participants provided their written informed consent to participate in this study. The animal study was reviewed and approved by Queen’s University Animal Care Committee.

## Author contributions

KZ conceived and conducted experiments, analyzed data, and wrote the manuscript. DS, JM, and HL conducted experiments. TC, OB, and BL contributed human patient samples. CT contributed reagents, conceived experiments, provided financial support. All authors read and edited the manuscript.
