# Supplementary material for: The dysregulation of leukemia inhibitory factor and its implications for endometriosis pathophysiology
Source: Front Immunol. 2023 Mar 23;14:1089098. doi: 10.3389/fimmu.2023.1089098 (PMC10076726; doi:10.3389/fimmu.2023.1089098)
Supplement: Supplementary file 1 [file DataSheet_1.docx]

Supplementary Material

The Dysregulation of Leukemia Inhibitory Factor and its Implications for Endometriosis Pathophysiology

Katherine B. Zutautas^1^, Danielle J. Sisnett^1^, Jessica E. Miller^1^, Harshavardhan Lingegowda^1^, Timothy Childs^1,2^, Bruce A. Lessey^3^, Olga Bougie^1,2^, Chandrakant Tayade^1*^

*** Correspondence:** Chandrakant Tayade: tayadec@queensu.ca

##
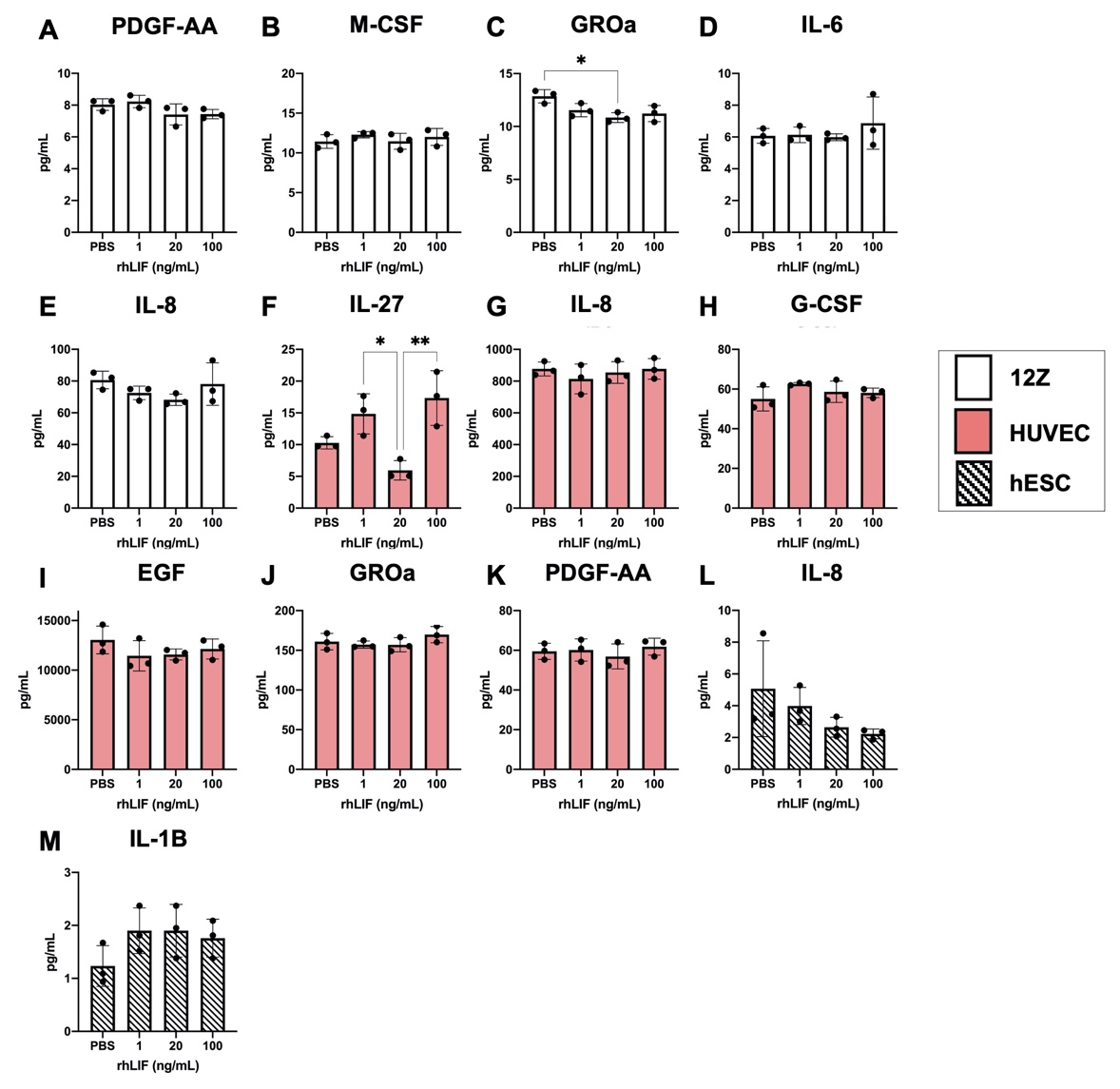
Supplementary Figures

**Supplementary Figure 1.** Cytokine analysis of cell supernatant from endometriosis representative cell lines treated with rhLIF. Cells- 12Z (**A-E**; white bars), HUVEC (**F-K**; red bars), and hESC (**L-M**; dashed bars) were treated for 24hrs with PBS or varying rhLIF concentrations (1, 20, 100ng/mL) and supernatant analyzed for 48 cytokines pertaining to angiogenesis, inflammation, and cell growth (HD48- Multi-plex Analysis, EveTech). Analysis performed as one-way ANOVA with Tukey post-hoc, *P<0.05, **P<0.01.
